# A Warning to White Slave Workers

**Published:** 1913-06

**Authors:** 


### A Warning to White Slave Workers.

We have every sympathy for the great effort being put forth to elevate moral standards and to alleviate the sufferings of victims of their own folly or precocious viciousness, but we think that purity workers generally will be the subjects of imposition if they are not hard-headed as well as soft-hearted. In a sense, nearly every human being is a slave. He or she gets started in a line of activity and, as time passes, it becomes harder and harder to change, while, in most instances, the daily demands are just about even with the daily means of supplying them. The lower socially, the more questionable morally the line of life work, the more nearly does it resemble a literal slavery. Yet, on reflection, it is plain that any successful opposition to pandering must be based on a very literal interpretation of white slavery and that any tendency to confusion with the moral, social and economic problems of prostitution in general will lead to difficulties. Among these difficulties may be mentioned the tendency to hysteria, fabrication and blackmail.

We feel that someone ought to make these remarks. Personally, we are skeptic as to the extent of white slavery, on the general economic ground of supply and demand. Why should anyone adopt the risky, expensive and elaborate methods of the white slaver when there is already a liberal supply of prostitutes and when men frequently boast that they never go near a house of prostitution, not as a matter of morality, but "because they can get all they want outside"? If it were conspicuous that white slavery represented an attempt to get immature girls or victims for perverts, we should take more stock in it. In a recent discussion of the subject one of our prominent physicians stated in rebuttal of the statement that every city had its white slave traffic, that he had formerly been employed by the health department in a capacity that led to his investigating most of the houses of prostitution of the city and that he had never seen a barred window nor come across other evidence of involuntary confinement.

Here is a true story, vouched for by a man from Chicago, of good standing and in close touch with the purity work of that city, that seems to indicate a need of the hard-headedness previously mentioned. A party of social workers went to a convention in another city. One of the active purity-workers—and our understanding is that he was actually an employed official in white slave cases—was accompanied by his wife. For the sake of economy they did not go to the hotel headquarters but sought a boarding house. They did not appear at the meeting the next day. After some search they were found in jail. The "boarding house" had been pulled in a police raid. They had told their story, which was absolutely true but so much stranger and more incredible than fiction that, naturally enough, the police had not accepted it. Now imagine a man, presumably grown up, since he had a wife, engaged in practical social work, perhaps having a voice in the conviction of other men for white slaving, so lacking in perspicuity. It reminds us of the story of the man who said to the farmer: "Will you kindly tell me what that very interesting plant is that you are cultivating?" The farmer replied: "By gum, I thought even a city man knew corn when he saw it—what's your business, stranger?" And the stranger replied that he was the editor of *The Practical Farmer*.

